# Prevalence of and risk factors for malaria, filariasis, and intestinal parasites as single infections or co-infections in different settlements of Gabon, Central Africa

**DOI:** 10.1186/s40249-017-0381-4

**Published:** 2018-01-30

**Authors:** Noé Patrick M’bondoukwé, Eric Kendjo, Denise Patricia Mawili-Mboumba, Jeanne Vanessa Koumba Lengongo, Christelle Offouga Mbouoronde, Dieudonné Nkoghe, Fousseyni Touré, Marielle Karine Bouyou-Akotet

**Affiliations:** 1 Department of Parasitology-Mycology, Faculty of Medicine, University of Health Sciences, P.O. Box 4009, Libreville, Gabon; 2 International Center for Medical Research of Franceville, Franceville, Gabon

**Keywords:** Malaria, Filariasis, Intestinal parasitic infections, Soil-transmitted helminths, Co-infection, Risk factors, Gabon

## Abstract

**Background:**

Malaria, filariasis, and intestinal parasitic infections (IPIs) are common and frequently overlap in developing countries. The prevalence and predictors of these infections were investigated in three different settlements (rural, semi-urban, and urban) of Gabon.

**Methods:**

During cross-sectional surveys performed from September 2013 to June 2014, 451 individuals were interviewed. In addition, blood and stool samples were analysed for the presence of *Plasmodium*, filarial roundworm, intestinal protozoan, and helminth infections.

**Results:**

Intestinal parasitic infections (61.1%), including intestinal protozoa (56.7%) and soil-transmitted helminths (STHs) (22.2%), predominated, whereas *Plasmodium falciparum* (18.8%), *Loa loa* (4.7%), and *Mansonella perstans* (1.1%) were less prevalent. Filariasis and STHs were mainly found in rural settlements, whereas a higher plasmodial infection prevalence rate was observed in the periurban area. The most common IPI was blastocystosis (48.6%), followed by ascaridiasis (13.7%), trichuriasis (11.8%), amoebiasis (9.3%), giardiasis (4.8%), and strongyloidiasis (3.7%). Hookworm was detected in one adult from rural Dienga. Adults had a higher prevalence of *Blastocystis hominis* and STHs, whereas *Giardia duodenalis* was more frequently observed among children aged below 5 years (*P* < 0.01). The polyparasitism rate was 41.5%, with 7.0% *Plasmodium-*IPIs and 1.8% *Plasmodium*-STH co-infections. The multivariate analysis showed that living in a suburban area, belonging to the age group of 5–15 years, having none or a secondary education, or having an open body water close to home were significant risk factors for malaria (*P* ≤ 0.01). For STH infections, identified risk factors were drinking untreated water and living in a rural area (*P* ≤ 0.04). No significant predictors were identified for IPIs and malaria-IPI co-infection.

**Conclusions:**

This study reports a high prevalence of IPIs and intestinal protozoa, but a low rate of malaria-IPI co-infections in the study sites. Improvements in the living conditions of the population such as adequate water supply and proper health education and sanitation should be integrated into control strategies for malaria, STHs, and IPIs.

**Electronic supplementary material:**

The online version of this article (10.1186/s40249-017-0381-4) contains supplementary material, which is available to authorized users.

## Multilingual abstracts

Please see Additional file 1 for translations of the abstract into the five official working languages of the United Nations.

## Background

Intestinal parasitic infections (IPIs), such as soil-transmitted helminth (STH) and protozoan infections, are recognized as major causes of illness and disease in disadvantaged communities. From 450 to 840 million cases occur worldwide, 95% of which are in developing countries [1].

Multiparasitism, defined as the simultaneous infection of a single host with two or more parasite species, is also common in individuals living in developing countries [2]. This term covers the simultaneous occurrence of IPIs and other communicable diseases, such as malaria and Human Immunodeficiency Virus (HIV), in the individual [3]. It has been suggested that co-infection may adversely affect outcome and influence host specific immunity [4, 5]. For example, chronic helminth infection leads to a modulation of the host immune response, with strong stimulation of the production of effector cytokines capable of downregulating the type 1 T helper cell response, thereby increasing vulnerability to other intracellular infections, such as malaria, HIV and tuberculosis [6, 7].

*Plasmodium*, the protozoan associated with the highest disease burden in Africa, may have a positive or negative impact on the progression of other parasitic diseases, without necessarily being affected itself [3, 8, 9]. In 2016, 216 million cases of malaria were reported worldwide [10]. In Gabon, malaria and IPIs are co-endemic, with prevalence rates ranging from 1% to 60% in rural and urban areas [5, 11–13].

*Plasmodium falciparum* infection is a common leading cause of consultation and hospitalization for febrile illness, and the number of clinical cases is currently increasing in the country [11, 13, 14]. Together with malaria and IPIs, loiasis is also a major cause of parasitic morbidity in the country [15, 16]. In addition, high prevalence rates of STH infections have been reported in rural settings, whereas data are scarce for the urban areas in which more than 60% of the population lives and where the water supply may be problematic [17]. This condition decreases the quality of life of poor and socioeconomically deprived populations and increases their exposure to IPIs. Recently, non-negligible prevalence of protozoa, such as *Cryptosporidium* spp*.* (10%) and *Giardia duodenalis* (20%), were recorded among asymptomatic children from a shantytown in Libreville, the capital city of Gabon [18].

All these pathogens are recognized as a public health problem in Gabon. Greater preventive efforts, such as mass drug administration (MDA) strategies and the development of effective vaccines, are urgently required. However, local reports on IPI prevalence and risk factors are scarce; those that do exist mainly focus on targeted species (primarily STHs) a single area, and/or specific age groups. Furthermore, polyparasitism and co-infections with other parasites, which seem to be more frequent than expected, are rarely studied.

Given the differences in IPI risk factors, such as socioeconomic status, environmental sanitation, level of education, access to safe water, climate, hygiene and nutritional status within regions, baseline infection levels should be recorded at different geographic sites, and the relationships between risk factors and parasite species and prevalence rates should be assessed. In Gabon, MDA with albendazole has recently been adopted and campaigns for the prevention of multiple parasitic infections will begin soon. However, baseline data estimating the real distribution and reservoirs of STHs and protozoan parasites are lacking, while different drugs are used for their elimination. Thus, the acquisition of such epidemiological and parasitological information should facilitate the design of integrated control programs for IPI prevention.

This study sought to investigate the epidemiology of malaria, single and multiple IPIs, and the risk factors associated with them, in three different settlements of Gabon.

## Methods

### Study sites

**Fig. 1:**
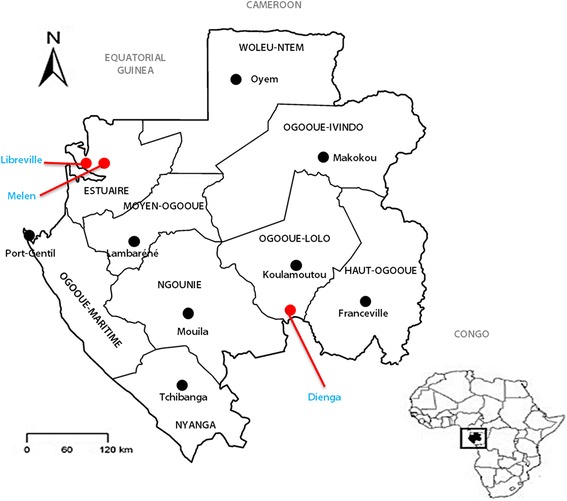
Study sites

A cross-sectional study was conducted from September 2013 to June 2014 in three areas of Gabon: Libreville, the capital city; Melen, a suburban town located 11 km from Libreville; and Dienga, a rural settlement of several villages located 779 km south-east of Libreville (see Fig. 1).

These three sites were selected because: one had a high probability of adult participation (Libreville), one had a high probability of children recruitment (Melen, which is also a crossroad between the capital city and rural areas), and one is remote settlement (Dienga).

Malaria prevalence was estimated at 24.1% for Libreville, 36% for Melen and 25.2% for Dienga [14, 19]. Malaria transmission in Gabon is perennial, with a slight peak of transmission during the rainy season [20]. The endemic blood filarial species in the country are *Loa loa* and *Mansonella perstans,* with prevalence rates of 22.4 to 39.5% in rural and urban regions of the country, respectively [15, 16].

### Sample size calculation

The sample size calculation was based on 5% precision with 95% level of confidence and an expected prevalence of STH infection of 7.9% [21]. This expected prevalence was based on the assumption that the prevalence of STHs, which was always found to be lower than the prevalence of protozoa, will be at least comparable to the recently reported prevalence (7.9%) of individuals living in Libreville [22]. Therefore, a minimum of 114 screened individuals was required. Consenting participants living in the study sites were prospectively included during the defined study period, which was estimated sufficient to include the required number of participants.

### Ethical considerations

This study was nested in a survey estimating the burden of microscopic and submicroscopic malaria in Gabon, which was approved by the National Ethic Committee for Research of Gabon (PROT N°0016/2013/SG/CNE for Libreville and Melen, and PROT N°0018/2013/SG/CNE for Dienga).

The study protocol and questionnaire were approved and registered under PROT No. 0011/2014/ SG/CNE and authorized by the Ministry of Health. Since participants at all sites had already given their written informed consent (or fingerprint if a participant was illiterate) for their participation in the main study evaluating the burden of malaria in Gabon and for data record and publication, it was agreed with the National Ethics Committee that their consent for stool examination and analysis of data will be given orally. Thus, oral informed consent was obtained from all adult participants and from the parents or guardians of the participating children.

The populations received information about the study from team members assisted by community workers who spoke the local language. The importance of the study for both personal and public health, its objectives, the procedures used, and the associated risks and benefits were explained to all participants. They were also informed that their personal information would be kept strictly confidential and they could withdraw from the study at any time without giving an explanation.

The main survey included free medical consultations for the participants and the administration of appropriate treatment according to national recommendations, with referral to an identified specialist when required. Biological testing was offered to participants and the results were supplied to their physicians for case management.

### Questionnaire

In each site, volunteers (adults, children with parents and/or guardians) were prospectively included during the study period. After consent had been obtained, each participant was questioned by a trained team member for the completion of a pre-tested questionnaire, which was based on the national Demographic and Health Survey.

Briefly, the questionnaire captured the following information: demographic characteristics (age, sex, marital status, education level); socioeconomic characteristics (occupation, type of house, income); behavioural factors (wearing shoes when outside, self-medication with antimalarial and/or anthelminthic drugs); and environmental and living conditions (type of drinking water, presence of toilets or a latrine, presence of water body close to home, presence and self-reported use of bed nets).

Participants had to have been living at the site for at least one month before the start of the study to be eligible for inclusion. For participants aged below 18 years and elderly participants under someone’s care, the parents and guardians or the heads of the family responsible for signing the informed consent form were interviewed.

### Biological sample collection

An amount of 5 ml of venous blood was collected into tubes containing ethylenediamine tetraacetic acid (EDTA), for the diagnosis of malaria and filariasis. The participants were also provided with clear instructions and clean well-labelled stool collection vials to ensure that stool samples were collected correctly. Each vial was identified by the code number of the participant.

The participant was instructed to use the scoop provided to transfer a thumb-sized faecal sample to the container, making sure that the sample was not contaminated with urine. Parents and guardians were advised to monitor their children during collection. Participants were asked to return the container the following day in the morning for rapid processing. Information on the exact collection time was not recorded.

### Blood sample examination

#### Malaria rapid diagnostic test (RDT)

A RDT (SD BIOLINE, SD Standard Diagnostics Inc., South Korea, Seoul) was performed according to the manufacturer’s instructions. This test allows the detection of *P. falciparum* and non-*falciparum* species. The results were communicated to the physicians for appropriate management.

#### Thick blood films

Thick films were used for microscopic diagnosis, as previously described [23]. They were dried and stained with 20% Giemsa stain for 20 min, then 100 × oil-immersion fields were read and parasitemia was determined as the number of parasites per microliter of blood. Smears were considered as negative if no parasite was detected on 100 × oil-immersion fields. Thin blood smears were used for species identification.

#### Detection of microfilariae

##### Direct microscopic examination

This was the first step for the microscopic detection and quantification of microfilariae in 10 μl of fresh, uncoagulated blood (collected into EDTA). Parasitemia was expressed as the number of microfilariae per milliliter (mf/ml) of blood.

##### Leukoconcentration technique

The second step was the realization of the Ho Thi Sang and Petithory technique, which requires 4 ml of blood, as previously described [24]. This technique has a higher sensitivity than the direct examination of total blood if parasite density is low. Because *M. perstans* parasitemia is often low, leukoconcentration was performed for all participants, regardless of their *L. loa* microfilaremia.

### Fecal sample examination

#### Direct microscopic examination

A small amount of the stool sample was mixed with a drop of saline (0.9% sodium chloride) and covered with a coverslip. The preparation obtained was examined under a light microscope for the observation of motile parasites. This method is used to detect STHs including *Schistosoma intercalatum* eggs, cysts, and vegetative forms of *Entamoeba* spp., *Blastocystis* spp., and *G. duodenalis*. The sample was considered negative if no intestinal protozoa or helminths were found on the entire slide.

#### MIF concentration

The merthiolate-iodine-formaldehyde (thimerosal) concentration (MIFc) method was carried out, as described by Sapero and Lawless [25]. The MIFc method is a simple, rapid staining and concentration technique, in which 0.25 g of the collected faecal specimen is concentrated in 2.35 mL thimerosal-formaldehyde solution and stained with 5% Lugol’s solution. *Ascaris lumbricoides* and *Trichuris trichiura* eggs, amoeba and *G. duodenalis* cysts and vegetative forms, and *Blastocystis hominis* cysts were stained. The stained preparation was homogenized and incubated for 30 min at room temperature. It was then filtered and centrifuged, and the entire pellet was used to generate a smear, which was examined under a light microscope.

The use of a combination of MIF and Lugol’s solution for staining is sensitive for the detection, fixation, and storage of vegetative forms and cysts of protozoa. The identification of protozoa is facilitated by the addition of iodine and eosin, which both stain vegetative forms bright yellow or light brown.

The species present were identified as follows. First, the labelled cyst or vegetative form was detected at a magnification of 10 × or 40 ×. It was centred on the microscope stage, a drop of immersion oil was added to the slide, and the parasite was identified by examining at a magnification of 1000 ×. The nuclear membrane is stained dark brown, the chromatin mass and siderophilic bodies are visible due to their refractive power, and free or phagocytized red blood cells appear bright red. Cysts are colourless against a red background. Parasite species observed after direct examination are also detected using the MIFc method which increases the detection or recovery of STH eggs. Samples were considered positive upon the detection of a single parasite.

#### Parasite culture

Parasites were cultured as previously described [26]. Briefly, about 1 g of stool sample was spread on a slide, covered with filter paper. This disposal was placed in a Petri dish and then sterile clean water was added in the bottom of the Petri dish. The preparation was incubated for 7 days at 25 °C. The water was collected, centrifuged, and the sediment was examined under a microscope on days three, five, and seven. *Necator americanus* and *Strongyloides stercoralis* larvae were detected. The sample was considered positive if at least one larva was present.

### Quality control of biological analysis

Two microscopists read all the slides for internal quality control.

### Definitions

- Presence of only intestinal helminths and no other parasite in a stool sample was considered a “single infection with helminths or single STH infection”.
- Presence of intestinal protozoa with no intestinal helminths was defined as a “single infection with intestinal protozoa”.
- The category of “other amoeba” comprises *Entamoeba hartmanni*, *Chilomastix mesnili*, and *Iodamoeba bütschlii*. They were considered non-pathogenic and were always associated with known pathogenic species such as *E. histolytica* or *G. duodenalis*.
- Presence of at least two different intestinal and/or blood parasite species was defined as “multiple parasite infections” or “polyparasitism”.
- The mean number of parasites per participant was defined as the mean number of parasites detected per infected participant.
- Unemployed meant not working for an income.

### Statistical analysis

Frequencies (%) of sociodemographic data of participants, and of the presence of *P. falciparum*, *L. loa*, *M. perstans*, intestinal parasites, STHs, intestinal protozoa, monoparasitism or multiple parasites (i.e. polyparasitism), *P. falciparum*/intestinal parasites, and *P. falciparum/L. loa* were determined. *P. falciparum, L. loa*, and *M. perstans* parasitemia are presented in medians with interquartile ranges (non-Gaussian distribution), while mean (± standard deviation) numbers of parasites are presented (Gaussian distribution).

Differences between the frequency of malaria and/or filariasis and/or IPIs, and comparison of the presence or absence of risk factors between and within groups (age groups and location) were assessed using chi-square or Fisher’s exact tests, if there were less than five expected values for proportions. The comparison of the mean number of parasite species according to age, sex, and location was carried out using the Student’s *t*-test or analysis of variance (ANOVA).

Crude odds ratios (c*OR*s) and 95% confidence intervals (*CI*s) were used to assess the association between malarial and intestinal parasite groups, and age, sex, education level, marital status, socioeconomic characteristics and polyparasitism variables. For all these tests, the difference was considered significant if *P* < 0.05.

All the reported *P*-values are for two-tailed tests. Logistic regression was performed to estimate the adjusted odds ratio (a*OR*) between malarial, intestinal parasite groups, and all variables with a *P* value <0.20 in the bivariate analysis. Because some groups size were very low (*n* < 10), a multivariate mixed logistic regression model using likelihood ratio estimation for discrete choice modelling of small datasets was performed. The a*OR*s and their 95% *CI*s were calculated. Associations were found significant if *P* values were below 0.05 and a trend was indicated if *P -*values were between 0.05 and 0.10. Stata version 13 (StataCorp, College Station, TX, USA) was used for data analysis. 

## Results

### General characteristics of the study population

Data from 451 consenting individuals were analysed, their blood samples were collected and analysed (see Additional file 2a). Intestinal parasite detection was performed using microscopic direct examination, MIFc, and parasite culture for 270 available stool samples, which were returned by the participants to the study team within the allocated time (see Additional file 2b).

According to the sociodemographic data, age was known for 402 participants, including 231 of those who provided stool samples. The age range was 3 months to 95 years, with children accounting for 63.2% of the study population. Just over half of the participants were female (51.2%).

Overall, 149 individuals were from rural Dienga, 179 from periurban Melen, and 123 from Libreville, the capital. Among the 361 adult participants, 36.8% were single. Overall, 35.9% had no education or did not complete primary school, with a higher proportion of this group in Dienga (61.5%) compared to Melen (22.6%) and Libreville (35.3%) (*P* = 0.01).

Almost two-thirds of the unemployed participants (59 out of 90) lived in Dienga. The subjects came from 269 households, with more than one family living in the same house in some cases. Most participants from Dienga (87.5%) lived in a wooden house. More than half of the participants from each site did not have modern toilets. A high proportion of those in Melen and Libreville drank tap water, whereas the same trend was observed for not wearing shoes when outside (64.1%) in Dienga. Overall, 177 of the 369 (48%) individuals for whom bed net information was available reported that they slept under a net (see Table 1).

**Table 1:** Characteristics of the study population, according to the study areas

	Urban	Suburban	Rural	Total
	n (%)	n (%)	n (%)	n (%)
Age group (*N*=402)				
0–4 years	32 (26.4)	80 (46.5)	19 (17.4)	131 (32.6)
5–15 years	22 (18.2)	83 (48.3)	18 (16.5)	123 (30.6)
>15 years	67 (55.4)	9 (5.2)	72 (66.1)	148 (36.8)
Gender (*N*=451)				
Female	70 (56.9)	81 (45.3)	80 (53.7)	231 (51.2)
Male	53 (41.1)	98 (54.7)	69 (46.3)	220 (48.8)
Marital status* (*N*=361)				
Single	38 (44.7)	45 (26.2)	50 (48.1)	133 (36.8)
In a couple	47 (55.3)	127 (73.8)	54 (51.9)	228 (63.2)
Education level* (*N*=351)				
No education	6 (7.1)	12 (7.1)	5 (4.8)	23 (6.6)
Primary school	24 (28.2)	26 (15.5)	59 (56.7)	103 (29.3)
Middle school	48 (56.5)	111 (66.1)	40 (38.5)	199 (56.7)
High school	7 (8.2)	19 (11.3)	0 (0.0)	26 (7.4)
Type of house (*N*=269)				
Brick house	38 (48.7)	44 (42.7)	4 (4.5)	86 (32.0)
Wooden house	37 (47.4)	50 (48.5)	77 (87.5)	164 (61.0)
Mixed house	3 (3.9)	9 (8.8)	7 (8.0)	19 (7.0)
Occupation (*N*=304)				
Middle manager	9 (15.3)	49 (29.9)	0 (0.0)	58 (24.4)
Senior manager	1 (1.7)	10 (6.1)	13 (17.6)	24 (3.6)
Employee	40 (67.8)	84 (51.2)	2 (2.7)	129 (42.4)
Unemployed	9 (15.3)	21 (12.8)	59 (79.7)	90 (29.6)
Type of toilet (*N*=269)				
Modern	32 (42.7)	82 (48.5)	20 (44.4)	114 (42.0)
Latrine	43 (57.3)	87 (51.5)	25 (55.6)	155 (58.0)
Self-reported net use (*N*=369)				
Yes	43 (46.2)	112 (64.7)	48 (73.9)	177 (48.0)
No	50 (53.8)	61 (35.3)	17 (26.1)	192 (52.0)
Open water body near home (*N*=293)				
Yes	36 (62.1)	116 (68.6)	0 (0.0)	152 (51.9)
No	22 (37.9)	53 (31.4)	66 (100.0)	141 (48.1)
Wearing of shoes when outside (*N*=147)				
Yes	55 (91.7)	102 (62.6)	37 (35.9)	80 (54.4)
No	5 (8.3)	61 (37.4)	66 (64.1)	67 (45.6)
Source of drinking water (N=271)				
Well	0 (0.0)	2 (1.2)	0 (0.0)	2 (0.7)
Spring	15 (20.0)	5 (2.9)	24 (96.0)	44 (16.2)
Tap	60 (80.0)	164 (95.9)	1 (4.0)	225 (83.1)

*Data recorded among adults and parents or guardians

### Plasmodial infection

**Fig. 2:**
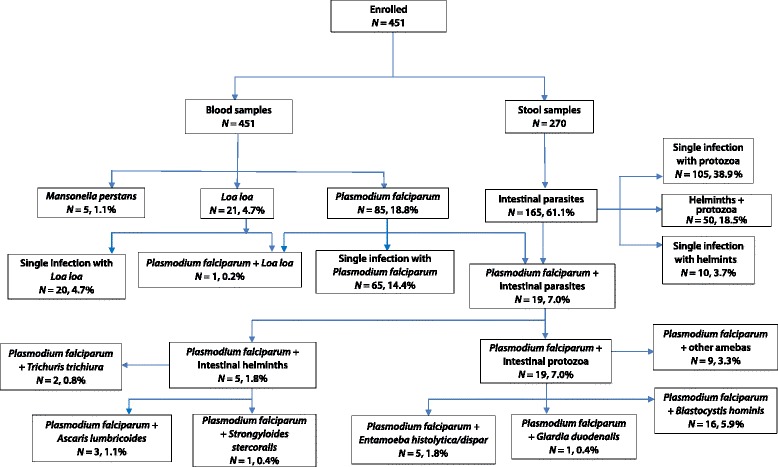
Prevalence and distribution of plasmodial infections, filariasis, and IPIs among the study participants

The only *Plasmodium* species identified was *P. falciparum*. The frequency of positive blood smears (PBSs) was 18.8% (85/451), with only three PBSs containing gametocytes associated with asexual forms (see Fig. 2 and Additional file 2a). The median parasite density was 7700 (287–36 400) trophozoites (T)/μl, and 54.1% (46/85) of the infected participants had a parasite count below 10 000 T/μl.

Of the Melen participants, 39.7% had a PBS, whereas less than 10% were infected in Libreville and Dienga (see Additional file 2a and Table 2). Children aged 5–15 years were more frequently infected by *P. falciparum* (*P* < 0.01) than the other age groups (see Table 2). Within age groups, children aged below 5 years from Dienga (33.3%; 5/15) were more likely to be infected than children from the same age group in Libreville (2.8%; 1/36) (*P* = 0.02).

**Table 2:** Distribution of parasite infections by age and location

	Age group, in years				Location			
	< 5	5–15	> 15	*P*	Urban	Suburban	Rural	*P*
	*n* (%)	*n* (%)	*n* (%)		*n* (%)	*n* (%)	*n* (%)	
*P. falciparum malaria* (*N* = 85)^a^	23 (17.6)	52 (42.3)	7 (4.7)	< 0.01	7 (5.7)	71 (39.7)	7 (4.7)	< 0.01
Blood filariasis (*N* = 26)^*b*^	1 (0.8)	1 (0.8)	17 (11.5)	–	6 (4.8)	0 (0.0)	20 (13.4)	–
Intestinal parasites (*N* = 165)^c^	23 (39.6)	28 (50.0)	86 (73.5)	< 0.01	50 (68.5)	29 (38.2)	86 (71.1)	< 0.01
Protozoan (*N* = 153)^d^	22 (37.9)	30 (53.6)	84 (71.8)	< 0.01	49 (67.1)	27 (35.5)	79 (65.3)	< 0.01
Helminths (*N* = 60)^e^	8 (13.8)	8 (14.3)	29 (24.8)	0.19	8 (11.0)	2 (2.6)	50 (41.3)	< 0.01
Polyparasitism (*N* = 112)^f^	15 (20.3)	22 (26.5)	55 (43.6)	< 0.01	7 (18.4)	9 (10.0)	76 (49.0)	< 0.01

^*a*^ *Ages known for 82 Plasmodium-infected participants*

^*b*^ *Ages known for 19 filariae-infected participants*

^*c*^ *Ages known for 137 intestinal parasite-infected participants*

^*d*^ *Ages known for 136 intestinal protozoa-infected participants*

^*e*^ *Ages known for 45 intestinal helminth-infected participants*

^*f*^ *Both ages and locations known for 92 participants with polyparasitism*

### Loiasis and blood mansonellosis

Overall, microfilariae were detected in 26 (5.8%) blood samples with 4.7% for *Loa loa* and 1.1% for *Mansonella perstans* (see Additional file 2a, Fig. 2, and Table 2). The median parasite density was 5500 (500–185 500) μf/ml for *L. loa* and 500 (500–1500) μf/ml for *M. perstans*. Eight (38.1%) of the 21 subjects with loiasis had microfilaria levels higher than 8000 μf/ml. No concomitant infection with *Loal loa* and *M.perstans* was detected.

Adult participants and those living in the rural area constituted the population at risk for blood filariasis: filariae were more frequently detected in adults (11.5%; 17/148) than in children (1.6%). Living in a rural area was associated with a higher risk of filarial infection, as 76.9% (20/26) of the infected individuals were from Dienga (*P* < 0.01) (see Table 2).

### Intestinal parasite infections

The results of faecal sample examination with MIFc and parasite culture are presented in Additional file 2b. The distribution of the intestinal parasite species detected is presented in Fig. 2, and Tables 2, 3 and 4. There was no detection of IPIs in 105 (38.9%) participants.

**Table 3:** Prevalence and distribution of parasite species, according to the study area

Parasite species, n (%)	Total (n=270)	Libreville (n=73)	Melen (n=76)	Dienga (n=121)
Blood parasites				
*P. falciparum*	37 (13.7)	3 (4.1)	31 (42.7)	3 (2.5)
Filariae	19 (7.0)	6 (8.2)	0 (0.0)	13 (10.7)
Intestinal Protozoa				
*G. duodenalis*	13 (4.8)	1 (1.4)	2 (2.6)	10 (8.3)
*E. histolytica/dispar*	25 (9.3)	4 (5.5)	3 (3.9)	18 (14.9)
*B. hominis*	131 (48.6)	40 (54.8)	26 (34.2)	65 (53.7)
*E. coli*	60 (22.2)	15 (20.5)	7 (9.2)	38 (31.4)
STHs				
*A. lumbricoides*	37 (13.7)	1 (1.4)	1 (1.3)	35 (28.9)
*T. trichiura*	32 (11.8)	2 (2.7)	1 (1.3)	29 (24.0)
*S. stercoralis*	10 (3.7)	5 (6.8)	0 (0.0)	5 (4.1)
*N. americanus*	1 (0.4)	0 (0.0)	0 (0.0)	1 (0.8)
Monoparasitism				
*P. falciparum*	19 (7.0)	1 (1.4)	18 (23.7)	0 (0.0)
Filariae	4 (1.5)	0 (0.0)	0 (0.0)	4 (3.2)
*A. lumbricoides*	2 (0.7)	0 (0.0)	0 (0.0)	2 (1.6)
*S. stercoralis*	1 (0.4)	0 (0.0)	0 (0.0)	1 (0.8)
*G. duodenalis*	1 (0.4)	0 (0.0)	0 (0.0)	1 (0.8)
*E. histolytica/dispar*	0 (0.0)	0 (0.0)	0 (0.0)	0 (0.0)
*B. hominis*	43 (15.9)	19 (26.0)	12 (15.8)	12 (9.9)
*E. coli*	6 (2.2)	4 (5.5)	1 (1.3)	1 (0.8)
Polyparasitism				
Two parasites	54 (20.0)	16 (21.9)	8 (10.5)	30 (24.8)
*P. falciparum* + intestinal protozoa	8 (3.0)	2 (2.7)	6 (7.9)	0 (0.0)
Filariae + intestinal protozoa	4 (1.5)	3 (4.1)	0 (0.0)	1 (0.8)
Filariae + STH	2 (0.7)	0 (0.0)	0 (0.0)	2 (1.6)
Intestinal protozoa	19 (7.0)	4 (5.5)	2 (2.8)	13 (10.7)
STH	5 (18.5)	1 (1.4)	0 (0.0)	4 (3.2)
Intestinal protozoa + STH	16 (5.9)	6 (8.2)	0 (0.0)	10 (8.3)
Three parasites	25 (9.3)	7 (9.6)	3 (3.9)	15 (12.4)
*P. falciparum* + intestinal protozoa	3 (1.1)	1 (1.4)	2 (2.6)	0 (0.0)
Filariae + intestinal protozoa	1 (0.4)	1 (0.4)	0 (0.0)	0 (0.0)
Filariae + STH + intestinal protozoa	4 (1.5)	0 (0.0)	0 (0.0)	4 (3.3)
Intestinal protozoa + STH	8 (3.0)	1 (1.4)	1 (1.3)	6 (5.0)
Intestinal protozoa	9 (3.3)	4 (5.5)	0 (0.0)	5 (4.1)
≥ Four parasites	33 (12.2)	2 (2.7)	5 (6.6)	26 (21.5)
*P. falciparum* + intestinal protozoa + STH	5 (1.5)	0 (0.0)	1 (1.3)	4 (3.2)
*P. falciparum* + intestinal protozoa	3 (1.1)	0 (0.0)	3 (3.9)	0 (0.0)
Filariae + STH + intestinal protozoa	1 (0.4)	0 (0.0)	0 (0.0)	1 (0.8)
Filariae + intestinal protozoa	3 (1.1)	2 (2.8)	0 (0.0)	1 (0.8)
Protozoa + STH	16 (5.9)	0 (0.0)	0 (0.0)	16 (13.2)
Protozoa	5 (1.8)	0 (0.0)	1 (1.3)	4 (3.2)

**Table 4:** Risk factors for *P. falciparum* infection

Parasite species, *n* (%)	< 5 years old (*n* = 58)	5–15 years old (*n* = 56)	> 15 years old (*n* = 117)	*P*
*E. histolytica/dispar*	2 (3.4)	5 (8.9)	9 (7.7)	0.46
*B. hominis*	20 (34.5)	26 (46.4)	70 (58.8)	<0.01
*G. duodenalis*	6 (10.3)	3 (5.4)	2 (1.7)	0.04
*A. lumbricoides*	5 (8.0)	5 (8.9)	15 (12.8)	0.01
*T. trichiura*	5 (8.0)	7 (11.1)	13 (12.5)	0.79
*S. stercoralis*	2 (3.4)	0 (0.0)	7 (6.0)	0.16
Hookworm	0 (0.0)	0 (0.0)	1 (0.9)	-

The prevalence of IPIs increased significantly with age: it was 39.6% in young children and 73.5% in participants over the age of 15 years (*P* < 0.01). Stool specimens from participants living in rural and urban areas were almost twice as likely to be infected as those obtained from the participants living in the suburban area (*P* < 0.01) (see Tables 2 and 3).

### Distribution of intestinal parasite species

Intestinal parasitic infections (57.4%, 155/270) were more common than STH infections (22.2%, 60/270) (*P* < 0.01) (see Table 3). Pathogenic species were found in 89.7% (139/155) of the infected samples. The highest prevalence recorded was for *B. hominis* (94.2%, 131/139), followed by *E. histolytica/dispar* (18.0%, 25/139) and *G. duodenalis* (9.4%, 13/139).

The global prevalence of gastrointestinal protozoa was higher among adults (71.8%) compared to the other age groups (53.6% and 37.9%) (*P* < 0.01) (see Table 2). In terms of the parasite species, *B. hominis* was more frequently detected in adults (*P* < 0.01), whereas *G. duodenalis* was mostly found in younger children (*P* = 0.04) (see Table 4). *Entamoeba coli*, *E. histolytica/dispar*, and *G. duodenalis* were more common in participants from the rural area than in participants from the suburban and urban areas (*P* < 0.01). There were more *B. hominis* infected samples in Libreville and Dienga (see Table 3).

The prevalence of STH infections among IPIs was 36.4% (60/165), with *A. lumbricoides* (61.7%, 37/60) the most frequently detected helminth, followed by *T. trichiura* (53.3%, 32/60), *S. stercoralis* (16.7%, 10/60), and hookworm, which was found only in one subject (1.7%, 1/60) (see Table 3). No *Schistosoma* species were detected.

Regardless of the parasite species, the rate of STH infections was highest among adults, who represented two-thirds of the STH-infected population of the rural city (*P* < 0.01) (see Table 4). Indeed, *A. lumbricoides* and *T. trichiura* were more common among adults and those living in Dienga (see Tables 3 and 4) (*P* < 0.01).

### Polyparasitism

Each infected participant had 1–6 different parasites. Infection with multiple parasites (blood and/or stool parasites) was common in the study population: 41.5% (112/270). The mean number of parasite species per participant was 1.43 (± 1.38); it was higher in females (1.6 ± 1.5) than in males (1.3 ± 1.2) (*P* = 0.02), in adults (1.6 ± 1.3) than in children aged 0–5 years (1.0 ± 0.8) (*P* < 0.01), and in participants living in the rural area (2.0 ± 1.7) than in the urban area (1.1 ± 1.0) (*P* < 0.01).

The frequency of single and multiple parasite infections was analysed in the population of the 270 participants who underwent blood and faecal examination. Polyparasitism predominated (41.5% versus the 28.1% single infection rate) when considering the whole population. In contrast, after stratification according to the study area, monoparasitism predominated in the suburban area of Melen (*n* = 31, 40.8% versus *n* = 16 multiple infected samples, 21.0%), whereas polyparasitism was common in rural Dienga (*n* = 71, 58.7% versus 17.4%, *n* = 21), while no difference was observed in the urban city (32.9% versus 34.2%). Double parasitism was more prevalent (see Table 3). There was no sample solely with a parasite of the “other amoeba” category or single *E. coli* infection.

### STH-IPI co-infections

The frequency of IPIs due to both STH and protozoa was 18.5% (50/270) (see Fig. 2 and Table 3). The most common combinations included *A. lumbricoides* (11.5%, 31/270); with *A. lumbricoides* plus *B. hominis* the most frequent combination recorded (38.0%, 19/50), followed by *A. lumbricoides* plus *T. trichiura* plus *B. hominis* (17.6%), and *A. lumbricoides* plus *E. histolytica/dispar* (13.7%). Indeed, *A. lumbricoides* was associated with at least two other parasites in 21 of the 31 samples. In addition, 8 of the 10 samples positive for *S. stercoralis* were also infected with *B. hominis*, with no other helminth present. Co-infections with *T. trichiura* plus *B. hominis* were found in 11 (21.9%) samples.

#### *Plasmodium falciparum* in association with microfilariae, IPIs, and STHs

Overall, 37 of the 85 *P. falciparum*-infected participants provided stool samples. Only one (1.2%) participant presented concomitant loiasis. All associations with *P. falciparum* and other parasites are shown in Fig. 2. The overall prevalence of *P. falciparum*-IPI co-infections was 7.0% (19/270).

All *Plasmodium*-infected participants with intestinal parasites harbored intestinal protozoa (see Table 3). The most common association was *P. falciparum* plus *B. hominis* (84.2%, 16/19), followed by *P. falciparum* plus “other amoeba” (47.3%, 9/19), and *P. falciparum* plus *E. histolytica/dispar* (26.3%, 5/19). *Plasmodium falciparum* was found in association with *G. duodenalis* only once (5.3%). Double parasitism, which included *P. falciparum*, comprised the combined infection *P. falciparum* plus *E. histolytica/dispar* only once and seven *P. falciparum* plus *B. hominis* infections*.*

Co-infection with STHs and *P. falciparum* was identified in five (13.5%) of the 37 individuals with a PBS, all five had concomitant intestinal protozoan infection. *P. falciparum* plus *S. stercoralis* and *P. falciparum* plus *T. trichiura* coparasitisms were each detected in one stool sample. A *P. falciparum* plus *A. lumbricoides* combined infection was detected in two stool samples. Polyparasitism with *P. falciparum* plus *A. lumbricoides* plus *T. trichiura* was observed in one stool sample.

The participant presenting co-infection with *P. falciparum* and *T. trichiura* lived in Libreville, whereas the others inhabited the rural area. All these subjects were below 15 years of age.

Among the other 233 malaria-negative participants who underwent stool examination, 140 (60.1%) had an IPI co-infection and 55 (23.6%) had at least one STH. There were similar numbers of participants with intestinal parasites in this group and among participants with a PBS (*P =* 0.32).

### Risk factors for parasitic infections

The bivariate analysis identified five factors associated with *P. falciparum* infection: belonging to the age group of 5–15 years (*P* < 0.01), not working (*P* < 0.01), living in the suburban area of Melen (*P* < 0.01), not using a bed net (*P* < 0.01), and not having taken any antihelminthic drugs in the last 12 months (*OR* = 1.29; 95% *CI* = 1.01–1.65; *P* = 0.038) (see Table 5).

**Table 5:** Risk factors for IPIs

	*P. falciparum* INFECTION			
Risk factors	c*OR* (95% *CI*)	*P*	a*OR* (95% *CI*)	*P*
Sex				
Female vs. male	1.2 (0.7–1.9)	0.53	1.2 (0.5–2.7)	0.7
Age group				
5–15 years vs. < 5 years	3.4 (2.0–6.2)	< 0.01	4.8 (1.9–13.6)	0.001
5–15 years vs. > 15 years	14.8 (6.8–37.1)	< 0.01	12.4 (2.7–95.7)	0.001
< 5 years vs. > 15 years	4.3 (1.9–11.1)	< 0.01	2.5 (0.5–20.7)	0.30
Marital status				
Couple vs. single	1.9 (1.1–3.3)	0.02	1.2 (0.5–3.4)	0.62
House type				
Wood vs. brick	0.7 (0.3–1.2)	0.19	0.8 (0.3–2.1)	0.72
Education level				
None vs. middle school	0.7 (0.4–1.2)	0.19	1.5 (0.5–4.3)	0.43
None vs. high school	0.7 (0.3–2.1)	0.48	8.3 (1.2–19.7)	0.03
Occupation				
Unemployed vs. employee	0.4 (0.2–0.7)	< 0.01	2.5 (0.6–10.7)	0.20
Unemployed vs. senior manager	0.5 (0.2–1.0)	0.05	1.7 (0.3–8.7)	0.54
Regular bed net use				
No vs. yes	7.6 (1.5–138.4)	< 0.01	1.6 (0.6–4.1)	0.29
Location				
Suburban area vs. rural area	5.9 (3.3–11.3)	< 0.01	5.9 (2.3–13.1)	< 0.01
Suburban area vs. urban area	3.7 (1.9–7.7)	< 0.01	2.8 (1.5–7.1)	< 0.01
Rural area vs. urban area	0.6 (0.3–1.4)	0.25	1.3 (0.7–4.6)	0.28
Antihelminthic drugs in the last 12 months				
No vs. yes	1.3 (1.01–1.65)	0.038	1.4 (0.4–6.3)	0.54
Open water body close to home				
Yes vs. no	1.5 (0.2–3.5)	0.12	2.9 (1.2–8.5)	0.048

Multivariate analysis by logistic regression confirmed that belonging to the age group of 5–15 years (*P* < 0.01), having no education (*P* = 0.03), living in a suburban area (*P* < 0.01), and having an open water body near home (*P =* 0.048) were significant risk factors for malaria (see Table 5).

The bivariate analysis showed that IPIs were associated with eight factors, including belonging to the age group of >15 years (four times higher odds of infection, *P* < 0.01), the head of the family having no or primary education (*P* < 0.01), being unemployed (*P =* 0.02), living in a rural area (*P* < 0.01), drinking water obtained directly from a river (*P* < 0.01) or spring (*P* < 0.01), using a latrine (*P =* 0.03) and having an open water body near home (*P* < 0.01) (see Table 6). Logistic regression analysis showed that only living in the rural area was a risk factor for IPIs.

**Table 6:** Risk factors for polyparasitism (bivariate analysis)

	IPI				STH infection			
Factors	c*OR* (95% *CI*)	*P*	a*OR* (95% *CI*)	*P*	cOR (95% *CI*)	*P*	a*OR* (95% *CI*)	*p*
Sex								
Female vs. male	1.2 (0.8–2.0)	0.4	0.7 (0.2–2.3)	0.55	1.4 (0.8–2.5)	0.29		
Age group								
5–15 years old vs. > 15 years old	0.4 (0.2–0.7)	< 0.01	0.7 (0.1–5.1)	0.68	0.5 (0.2–1.1)	0.11	0.5 (0.5–2.3)	0.49
> 15 years old vs. < 5 years old	4.2 (2.2–8.3)	< 0.01	5.1 (0.4–67.5)	0.19	2.1 (0.9–5.1)	0.08	2.7 (0.9–4.4)	0.09
< 5 years old vs. 5 – 15 years old	0.7 (0.3–1.4)	0.27	0.3 (0.04–1.6)	0.17	1.0 (0.3–2.8)	0.94		
Marital status								
Couple vs. single	0.6 (0.3–1.0)	0.07	2.7 (0.7–11.8)	0.15	0.7 (0.4–1.4)	0.34		
House								
Wood vs. brick	1.7 (0.8–3.9)	0.19	0.9 (0.2–4.7)	0.91	6.4 (1.8–41.4)	< 0.01	2.9 (0.4–27.8)	0.27
Education level								
None vs. middle school	6.0 (1.7–27.8)	< 0.01	2.3 (0.6–11.3)	0.30	2.3 (1.1–4.7)	0.02	2.1 (0.8–4.1)	0.18
None vs. high school	5.0 (1.5–23.2)	< 0.01	3.2 (0.1–125.6)	0.48	5.5 (1.0–102.7)	0.04	3.3 (0.7–121.1)	0.66
Occupation								
Unemployed vs. employee	1.6 (0.8–3.5)	0.19	0.5 (0.05–4.7)	0.58	6.6 (2.3–24.3)	< 0.01	6.6 (0.9–16.5)	0.07
Unemployed vs. senior manager	2.7 (1.1–6.7)	0.02	0.8 (0.08–7.4)	0.84	3.7 (1.3–14.0)	0.02	0.9 (0.06–12.9)	0.87
Regular bed net use								
No vs. yes	0.6 (0.3–1.1)	0.09	0.7 (0.1–2.7)	0.57	2.7 (1.1–7.3)	0.03	1.2 (0,18–7.3)	0.85
Location								
Rural area vs. suburban area	4.7 (2.4–9.3)	< 0.01	4.4 (1.9–9.0)	<0.01	14.1 (4.2–28.6)	< 0.01	14.0 (4.1–13.5)	< 0.01
Rural area vs. urban area	2.9 (1.3–6.3)	< 0.01	2.7 (1.1–5.8)	0.03	4.3 (2.6–8.9)	< 0.01	3.2 (2.0–7.9)	< 0.01
Drinking of river water								
Yes vs. no	4.9 (1.5–22.5)	< 0.01	3.4 (0.8–10.5)	0.18	10.2 (2.9–28.5)	< 0.01	8.1 (1.4–18.6)	0.03
Drinking of spring water								
Yes vs. no	4.1 (1.5–13.4)	0.01	3.1 (1.0–10.7)	0.06	5.5 (1.7–17.3)	<0.01	3.6 (1.1–22.2)	0.04
Use of latrines								
Yes vs. no	2.8 (1.1–7.7)	< 0.01	2.1 (0.4–11.2)	0.35	12.8 (1.5–26.7)	0,02	9.9 (0.8–13.3)	0.51
Open water body near home								
Yes vs. no	2.4 (1.2–4.7)	0.01	0.4 (0.05–2.6)	0.13	14.0 (3.9–89.3)	<0.01	9.1 (1.2–17.0)	0.02
Wearing of shoes when outside								
No vs. Yes	1.8 (0.9–3.4)	0.09	0.9 (0.2–4.04)	0.9	2.7 (1.1–7.3)	0,03	1.4 (0.2–12.1)	0.76

The bivariate analysis determined that STH infections were associated with living in a wooden house (*P* < 0.01), having no education (*P* < 0.01), being unemployed (*P* = 0.01), not using a bed net (*P* = 0.03), living in a rural area (*P* < 0.01), drinking untreated water (*P =* 0.03), using latrines (*P* = 0.02), having an open water body near the home (*P* = 0.03), and not wearing shoes when outside (see Table 6). Having an open water body near home (*P* = 0.02), drinking untreated water from a river or spring (*P* = 0.03), and living in a rural area remained significantly associated with STH infections after the multivariate analysis was conducted (Table 6).

A bivariate analysis was also carried out for *P. falciparum*-IPI co-infections. For such co-infections, belonging to the age group of 5–15 years (*P* = 0.02), having an occupation (*P* = 0.04), living in a suburban area (*P* = 0.02), and drinking water from a river or spring (*P* = 0.04) were identified as risk factors (see Table 7). None of the studied variables remained significantly associated with polyparasitism after logistic regression analysis was conducted (data not shown).

**Table 7:** Factors associated with single or co-infections. Results of the bivariate analysis with polyparasitism as the outcome

Factors	COR [95% *CI*)	*P*
Sex		
Female vs. male	1.4 [0.6–4.0)	0.46
Age group		
5–15 years vs. < 5 years	3.7 [1.2–13.9)	0.02
5–15 years vs. > 15 years	15.7 [4.1–103.4)	<0.01
< 5 years vs. > 15 years	4.3 [0.8–31.4)	0.09
Marital status		
Couple vs. single	1.6 [0.6–5.2)	0.39
House type		
Wood vs. brick	0.5 [0.1–2.0)	0.29
Education level		
None vs. middle school	0.4 [0.1–1.3)	0.12
None vs. high school	0.7 [0.1–13.2)	0.72
Occupation		
Unemployed vs. employee	0.2 [0.0–0.9)	0.04
Unemployed vs. senior manager	0.3 [0.0–1.5)	0.13
Regular bed net use		
No vs. yes	2.6 [0.9–8.8)	0.08
Location		
Suburban area vs. rural area	4.0 [1.2–14.0)	0.02
Suburban area vs. urban area	0.8 [0.2–3.1)	0.79
Rural area vs. urban area	0.2 [0.1–0.8)	0.02
Drinking river water		
Yes vs. no	4.1 [1.0–19.8)	0.06
Drinking spring water		
Yes vs. no	2.7 [0.5–51.1)	0.28
No vs. yes	1.1 [0.3–5.3)	0.86
Use of latrines		
Yes vs. no	5.6 [1.7–25.6)	<0.01
Open water body near home		
Yes vs. no	0.9 [8.8–0.1)	0.08
Wearing shoes when outside		
Yes vs. no	0.9 [8.8–0.1)	0.08

## Discussion

In this study, the epidemiology of malaria, filariasis, and IPIs were investigated in three areas of Gabon. While data on pregnant women and children already exist, those on non-pregnant adults and from populations living in urban cities were lacking.

More than half of the study participants tested positive for at least one parasite (55.4%). As positivity was based on the results of only one stool and/or blood sample and not all 451 individuals underwent complete parasitological analysis, we would expect the true rates of infection in the population to be higher. Indeed, asymptomatic polyparasitism is now often described in Africa [8, 9, 24]. Although the prevalence rate of parasite carriage (61.1%) in this study is lower than those previously reported in pregnant women and children from rural areas of Gabon (90.0%), the burden of parasitic diseases is non-negligible in this asymptomatic population [17].

*Plasmodium falciparum* infection was found in only 18.8% of participants. The asymptomatic carriage may account for this lower prevalence rate than that reported in recent studies conducted in Gabon among febrile participants (38.0% in 2011 and 42.0% in 2012) [11, 14]. Gabon is experiencing an epidemiological transition, with the prevalence of clinical malaria currently on the increase, following a significant decline after the introduction of artemisinin-based combination therapies, and intermittent preventive treatment [13, 14, 20]. A similar trend is being observed for rates of asymptomatic *P. falciparum* infection, which were 12.6% in 1991 and 12.0% in 2004, then estimated at 6.2% in several villages of the country in 2008, with rates of 13.0–26.0% reported since 2014 [19, 27–29].

Older children (5–15 years of age) were four times more likely than younger children to be infected with malarial parasites, as reported in another setting, involving a change from hyper- to meso-endemicity [30]. Nevertheless, in Dienga (rural area), children under the age of five were more frequently infected than children of the same age from Libreville (urban area). This trend in malaria risk seems to apply to all age groups and transmission rates remain high in rural areas of Gabon in which access to control strategies is limited [14, 19, 31]. This study’s results give incentives for improved malaria control in rural areas and also confirm the heterogeneity of malaria endemicity in Gabon.

The prevalence of IPIs was 61.1%, a value greater than the 49.0% reported for Lambaréné, another rural area located 229 km from the capital city. Reports on IPIs, which include protozoa and association with filarial infections and malaria, are scarce in neighbouring countries with comparable environmental and sociodemographic profiles. Thus, in comparison with other settings, the observed frequency of intestinal parasitism in the asymptomatic population is higher than the 28.6 and 11.9% reported respectively in Muyuka and Mfou districts in Cameroon and the 33.2% recorded in Côte d’Ivoire [8, 9, 32]. Higher prevalence rates have been found in Ethiopia (81.0%) and Malaysia (98.4%) [33, 34]. Intestinal parasitism was more prevalent in older children and adults, regardless of the parasite species, consistent with findings of other studies [9, 32, 33, 35]. Thus adults constitute a non-negligible parasite reservoir likely to contribute to contamination within the family and in the professional environment.

The rates of infection with STHs and intestinal protozoa were 22.2 and 56.7%, respectively. The higher prevalence of intestinal protozoa than of helminths is consistent with recent reports from Kenya and Tajikistan [36, 37]. Helminth species predominate in rural areas, as previously reported in Gabon and elsewhere [6, 17]. In Cameroon and Côte d’Ivoire, STH infections also predominate in rural areas [8, 32]. Low socioeconomic status, poor living conditions, insufficient knowledge and practice of correct sanitation, and limited access to safe water, which are known risk factors for STHs and IPIs, are also common in populations from these settlements [7, 9, 30, 32–36]. Indeed, people not having education and being unemployed, people drinking unsafe water, not wearing shoes when outside, and using latrines, particularly if inappropriately maintained and the presence of an open water body close to people’s dwellings are all common in the rural area of Dienga. These factors were also found to be significantly associated with STHs. Furthermore, the rates of STHs increased over the gradient from urban to rural areas, regardless of the parasite species. This finding is consistent with reports from Ethiopia (70.8%in rural area versus 5.2% in a urban city) and Rwanda (54.5% in a remote area versus 20% in a periurban area), but contrasts with findings from Côte d’Ivoire, Rwanda and Malaysia [33–38].

The predominant STH species were *A. lumbricoides* and *T. trichiura*, as reported in remote areas of Cameroon and Malaysia [8, 9, 39]. Hookworm and *S. stercoralis* were predominantly found in adults from Dienga, where risk factors such as the presence of an open water body near the home, poorly maintained latrines, and not wearing shoes when outside are frequent. Both these parasites are transmitted via skin penetration, which is favoured by these conditions. In 2012, a lower prevalence (20.2%) of STH infections was also reported in a population of older children living in Plaine-Oréty, a neighborhood of Libreville, while studies performed in Gabon more than 20 years ago reported a higher prevalence of helminth infections; this difference may be due to self-medication with albendazole, which is frequently prescribed or provided to participants presenting digestive symptoms, whatever the etiology [40]. Likewise, in Cambodia, the prevalence of intestinal helminth infections decreased after 2006, the year in which the MDA of mebendazole began [41]. On the other hand, the higher prevalence of protozoan infections in this study may be explained by the systematic prescription of an antihelminthic drug or deterioration in the living conditions of the populations of Libreville and surrounding areas. Indeed, Libreville and Melen have experienced unplanned urban expansion, with slums and shantytowns creating environmental conditions, such as poverty, poor environmental sanitation, and lack of clean water, which are likely to favour IPIs. With the frequent shortage of clean drinking water observed in these cities, communal water supply points have been established, increasing the risk of contamination during the transport and storage of drinking water from its source to its point of use. Interestingly, species associated with poor hygiene and an inadequate water supply, such as *E. coli* and *B. hominis*, predominate in the study population. Nevertheless, the MIFc method used for parasite detection may also have contributed to the high frequency of protozoa recorded here, whereas in other studies, methods used for faecal examination could be also less sensitive for the detection of amoeba cysts and vegetative forms, or the protozoan infection status was not the main purpose of the examination.

The pathogenic protozoa *G. duodenalis* and *E. histolytica/E. dispar* were detected in lower proportions of asymptomatic subjects. All these protozoa, including the suspected pathogenic *B. hominis*, may cause infections that remain asymptomatic and thus undiagnosed for long periods, rather than those that tend to be chronic. Furthermore, the observed different age distribution of *B. hominis* and *G. duodenalis* is in agreement with previous reports [42, 43]. Such infections, when becoming chronic, can have a deleterious impact on normal physical and cognitive development, highlighting the necessity of treating asymptomatic individuals and preventing chronic infections.

Polyparasitism is a marker of poor sanitation and poverty, but it is also associated with increased susceptibility to other diseases such as malaria and impairment of nutritional anemia [4–6, 39]. It was common (41.5%) in the study population and mostly involved co-infections with different intestinal protozoa. Rates of *P. falciparum-*intestinal protozoa (11.5%) or *P. falciparum*-IPI (7.0%) co-infection were lower than those reported in Côte d’Ivoire (18–19%) and rural Cameroon (22.1%), while prevalence rates of 5.0–11.0% have been recorded in Tanzania, Ethiopia, and other cities in Cameroon [8, 9, 30, 32, 42, 43]. These dissimilarities may be explained by differences in living conditions (rural versus urban areas) and population characteristics (febrile versus asymptomatic *P. falciparum*-infected participants). However, it is difficult to draw conclusions about the associations between *P. falciparum* infection and IPIs, as most plasmodial infections were found in the suburban city of Melen in children who were less likely to have IPIs, whereas most IPI-STH infections were found in adults and rural areas with low malaria frequencies. Further studies evaluating the burden and intensity of protozoan and STH co-infections in the *P. falciparum*-infected population will provide valuable information about the relationship and the impact of co-infections on the progression and outcome of each parasitic infection.

The prevalence rates of *L. loa* and *M. perstans* infections were low (5.8%), contrary to previous data of other cities in Gabon, in which at least 20% of the individuals screened carried one or both of these worms [15, 16]. The participants of the present study came from urban, periurban, and rural areas, and almost two-thirds were children. It is known that due to their daily activities, such as farming (the main activity in rural areas), adults are more susceptible to carry filariae. The predominance of loiasis in Dienga, with a prevalence similar to that found previously, corroborates this [44].

Regardless of the parasite and type of sample (blood or stool), the prevalence of infections increased with age in our study. Older children and adults were found to be three and five times more likely, respectively, than younger children to be infected, consistent with most recent studies performed in tropical areas, whereas younger children were more likely to have polyparasitism [8, 9, 33, 45]. Behavioural factors, outdoor activities, and a lower frequency of self-medication may account for this link between age and parasite burden, at least for IPIs. It is obvious that older children and adults constitute a major parasite reservoir and these groups should therefore be targeted, in addition to preschool children, in programs to control malaria and IPIs. Drugs targeting intestinal protozoa, which are certainly deleterious in cases of chronic infections, should be investigated to determine the feasibility of integrating such drugs into mass deworming campaigns.

This is the first study to provide data on the risk factors for single and multiple parasite infections in Gabon. The key findings were that age, and factors such as being unemployed (indicating a low family income) and proximity to an open water body were associated with a prevalence of malaria and IPIs. The small number of *P. falciparum-*infected individuals and the similar levels of self-reported bed net use between sites and age groups may account for the absence of a relationship between malaria and bed net use in the multivariate analysis. Consistent with the findings of many reports, inadequate general and personal hygiene, an unsafe water supply, low levels of parental education, and being unemployed have been associated with polyparasitism, particularly with STHs [33–36, 38, 40]. Parasite species-specific risk factors were not investigated, but the factors cited above, together with the contamination of drinking water during its transport from dams or wells to houses and its storage in containers, were also found to be significantly associated with the presence of *Giardia*, *E. histolytica*, and *B. hominis*, the predominantly detected species in the present study [3, 6, 46–48]. All these conditions are common in rural areas and have also been common in the urban areas of Gabon over the last 5 years. Indeed, frequent drinking water shortages have led to the establishment of communal supply points, increasing the risk of population contamination. Beside all these factors, household conditions and exact composition of families, close contact within families and domestic animals, and frequency of hand washing after defecation and before eating, which were not investigated here, are significantly associated with IPIs [33, 34, 38, 45]. Therefore, together with other control strategies, health education of all age populations, which has been abandoned for more than 15 years, should be re-implemented in the country.

This study has several limitations. Stool samples were collected only once from the subjects, potentially accounting for the lower prevalence of intestinal helminths than of intestinal protozoa. The intensity of IPIs was not determined. However, the sensitivity of MIF staining and concentration has been shown to be high for the detection of common STHs and protozoan species, including *Schistosoma* species, even though the Kato-Katz method is usually used for the detection and quantification of *S. mansoni* in the field. Neither this species nor *Taenia* spp*.* are endemic in Gabon. Data for the prevalence of *Cryptosporidium* spp. and *Isospora belli* infections were provided in a previous report [18]. Nevertheless, the study provides local data on IPIs prevalence and human reservoir, on malaria-IPI co-infections and on associated risk factors. These data are important for the design or the readjustment of control strategies for the prevention of these infections, at the time of the recent introduction in Gabon of MDA with albendazole which only targets STH.

## Conclusions

Intestinal parasite infections are highly prevalent and together with malaria are a major public health concern in the poor and socioeconomically deprived communities of the urban and rural areas of Gabon. The burden of IPIs in this study was surprisingly high. Additional investigations in other parts of the country are now required. Integrated control strategies focusing on better health education and improvements in environmental sanitation and hygiene, coupled with improved housing, access to safe water, and distribution of ITNs should be developed and implemented in Gabon.

## Additional files

Additional file 1: Multilingual abstracts in the five official working languages of the United Nations. (PDF 241 kb)

Additional file 2: a) Number of blood samples per site and positive results according to diagnostic methods; b) Number of stool samples per site and positive results according to diagnostic methods. (DOCX 102 kb)

## Abbreviations

CI: Confidence interval
COR: Crude odds ratio
EDTA: Ethylenediamine tetraacetic acid
HIV: Human immunodeficiency virus
IPI: Intestinal parasitic infection
ITN: Insecticide-treated net
MDA: Mass drug administration
MIFc: Merthiolate-iodine-formaldehyde concentration
PBS: Positive blood smear
RDT: Rapid diagnostic test
STH: Soil-transmitted helminth
