# Correction to: prevalence of and risk factors for malaria, filariasis, and intestinal parasites as single infections or co-infections in different settlements of Gabon, Central Africa

**DOI:** 10.1186/s40249-018-0415-6

**Published:** 2018-04-20

**Authors:** Noé Patrick M’bondoukwé, Eric Kendjo, Denise Patricia Mawili-Mboumba, Jeanne Vanessa Koumba Lengongo, Christelle Offouga Mbouoronde, Dieudonné Nkoghe, Fousseyni Touré, Marielle Karine Bouyou-Akotet

**Affiliations:** 1 Department of Parasitology-Mycology, Faculty of Medicine, University of Health Sciences, P.O. Box 4009, Libreville, Gabon; 2 International Center for Medical Research of Franceville, Franceville, Gabon

## Correction

Unfortunately, the original article [1] contained some errors. The table title of Tables 4, 5, 6, 7 were interchanged by mistake and displayed incorrectly in the article. The correct table titles of Tables 4, 5, 6, 7 can be found below.

Table 4: Distribution of intestinal parasite species, according to the age.

Table 5: Risk factors for *P. falciparum* infection.

Table 6: Risk factors for IPIs.

Table 7: Risk factors for polyparasitism (bivariate analysis).
