# Supplementary material for: Prevalence of and risk factors for malaria, filariasis, and intestinal parasites as single infections or co-infections in different settlements of Gabon, Central Africa
Source: Infect Dis Poverty. 2018 Jan 30;7:6. doi: 10.1186/s40249-017-0381-4 (PMC5789590; doi:10.1186/s40249-017-0381-4)
Supplement: Supplementary file 1 — Multilingual abstracts in the five official working languages of the United Nations. (PDF 241 kb) [file 40249_2017_381_MOESM1_ESM.pdf]

Translation of the abstract into the five official working languages of the United Nations

تفشي الملاريا وداء الخيطيات (فيلاريسيس) والطفيليات المعوية وعوامل الخطر التي تؤدي إليها كحالات عدوى منفردة أو التهابات مشتركة في أماكن مختلفة في الغابون، في أفريقيا الوسطى

نوي باتريك مابوندوكوي، ايريك كيندجو، دينيس باتريشيا ماولي مابومبا، جين فانيسا كومبا لينغونغو، كريستل اوفوغا مابوروند، ديودون كو، فاوسيني توري، ماريل كارين باويو أكوتيل.

ملخص الدراسة

خلفية الموضوع: تعد الملاريا وداء الخيطيات (فيلاريسيس) والعدوى الطفيلية المعوية شائعة وغالباً ما تتداخل في البلدان النامية. وقد تم البحث بموضوع تفشي هذه العدوى وتوقع الإصابات بها في ثلاث مستوطنات مختلفة (الريفية وشبه الحضرية والحضرية) من الغابون.

منهجية البحث: تم مقابلة 451 شخصاً خلال جمع الاستبيانات التي شملت عدة قطاعات والتي تمت مابين شهر سبتمر 2013 و شهر يونيو 2014. بالإضافة إلى ذلك، تم تحليل عينات من الدم والبراز للتأكد من وجود الملاريا (المتصورة النشيطة)، الدودة الخيطية، البروتوزوان المعوية، والتهابات الديدان الطفيلية.

النتائج: بلغت نسبة الإصابة بالعدوى الطفيلية المعوية بنسبة (61.1٪)، بما في ذلك البروتوزوا المعوية بنسبة (56.7٪) والديدان المنقولة عبر التربة بنسبة (22.2٪)، في حين بلغت نسبة البلازمود المنجلي (18.8٪)، وعدوى لوا لوا (4.7٪)، ونسبة المَنْسُونيلَّةُ اللَّجُوجَة (1.1٪) وهي الأقل انتشارا. ووجد أن داء الخيطيات (الفيلاريات) والديدان المنقولة عبر التربة تتواجد أساساً في الأماكن الريفية، في حين لوحظ ارتفاع معدل انتشار العدوى البلازودية (المتصورة النشيطة) في منطقة الضواحي. وكان أكثر أنواع العدوى الطفيلية المعوية شيوعاً هو الأكياس الأريمية بنسبة (48.6٪)، يليها داء الإسكارس بنسبة (13.7٪) وداء المسلكات أو "داء شعريات الذيل" بنسبة (11.8٪) و داء الأميبات (9.3٪) و عدوى الجيارديا بنسبة (4.8٪) وداء الاسطوانيات بنسبة (3.7٪). وقد تم الكشف عن الدودة الشصية في شخص بالغ واحد من الريف في دينغا. وكانت نسبة الأكياس الأريمية و الديدان المنقولة عبر التربة هي الأكثر شيوعاً بين البالغين. في حين لوحظ أن الجيارديا المعوية كانت الأكثر شيوعاً بين الأطفال الذين تقل أعمارهم عن خمس سنوات بنسبة ($P < 0.01$).و كان معدل متعددة الطفيليات بنسبة 41.5٪، بنسبة 7.0٪ للعدوى البلازودية (المتصورة النشيطة) والعدوى الطفيلية المعوية ونسبة 1.8٪ للعدوى البلازودية (المتصورة النشيطة) وعدوى الديدان المنقولة عبر التربة. وأظهر تحليل متعدد المتغيرات أن أولئك الذين يعيشون في منطقة الضواحي ضمن الفئة العمرية من 5 إلى 15 سنة وليس لديهم أي تعليم أو تعليم ثانوي، أو أن يكون لديهم مياه مفتوحة في مكان قريب من المنزل، كانت عوامل خطر الإصابة بمرض الملاريا أكبر بنسبة ($P \le 0.01$). أما بالنسبة للإصابة بالعدوى المنقولة عبر التربة فإن عوامل الخطر التي تم تحديدها هي شرب المياه غير المعالجة والعيش في منطقة ريفية بنسبة ($P \le 0.04$) . ولم يتم تحديد أي عوامل هامة لعدوى الطفيليات المعوية وعدوى الملاريا والطفيليات المعوية المشتركة.

الخاتمة: تشير هذه الدراسة إلى ارتفاع معدل انتشار عدوى طفيليات الأمعاء و بروتوزوا الأمعاء، ولكن نسبة منخفضة من الملاريا كانت الإصابة فيها بسبب الديدان المنقولة عبر التربة والالتهابات المشتركة في مواقع الدراسة. وينبغي إجراء تحسينات في الظروف المعيشية للسكان، مثلاً الإمداد الكافي للمياه والتثقيف الصحي والنظافة ودمجها في استراتيجيات مكافحة الملاريا وعدوى الديدان المنقولة عبر التربة والعدوى بالطفيليات المعوية.

Translated from English version into Arabic by Randa82, through

TRANSLATORS WITHOUT BORDERS

非洲中部加蓬的不同定居点疟疾、丝虫病和肠道寄生虫单纯感染或混合感染的流行和危险因素

Noé Patrick M'bondoukwé, Eric Kendjo, Denise Patricia Mawili-Mboumba, Jeanne Vanessa Koumba Lengongo, Christelle Offouga Mbouoronde, Dieudonné Nkoghe, Fousseyni Touré, Marielle Karine Bouyou-Akotet

摘要

引言：疟疾、丝虫病和肠道寄生虫感染(IPIs)在发展中国家非常普遍，且会同时出现。本研究在加蓬3个不同的定居点(农村、半城市和城市)对上述感染的发病率和预测因子进行了调查。

方法：在2013年9月至2014年6月进行的横断面调查中，共计451人接受访问。此外，研究人员还对血液和粪便样本进行了分析，以期发现疟原虫、丝线虫、肠道原虫和蠕虫感染。

结果：寄生虫感染以肠道寄生虫感染(61.1%)为主，包括肠道原虫(56.7%)和土源性蠕虫(STHs)(22.2%)。而恶性疟原虫(*Plasmodium falciparum*，18.8%)、罗阿丝虫(*Loa loa*，4.7%)和常现曼森氏线虫(*Mansonella perstans*，1.1%)流行程度较低。丝虫病和STHs主要见于农村居民点，而城郊地区的疟原虫感染率较高。最常见的IPI是芽囊原虫病(48.6%)，其次是蛔虫病(13.7%)、鞭虫病(11.8%)、阿米巴病(9.3%)、贾第虫病(4.8%)，和粪圆线虫病(3.7%)。在Dienga村的一个成人体内发现钩虫。成人的人芽囊原虫感染和STHs的患病率较高，而在5岁以下的儿童中十二指肠贾第鞭毛虫（*Giardia duodenalis*）感染更为常见 ($P < 0.01$)。多重感染率为41.5%，其中7.0%的为疟原虫和IPIs合并感染，1.8%的为疟原虫和STH合并感染。多变量分析表明，生活在郊区，年龄介于5 ～ 15岁，未接受过中等教育，或居所附近有开放水体是罹患疟疾的重要危险因素($P \leq 0.01$)。对于STH感染，饮用未经处理的水和生活在农村地区 ($P \leq 0.04$) 是可识别的风险因素。对IPIs和疟疾-IPI混合感染没有显著的预测因子。

结论：本研究报告了较高的IPIs和肠道原虫患病率，但在调查点中，疟疾-IPI混合感染的发生率较低。改善人群生活条件，如充足的水源供应、适当的卫生教育和卫生设施应纳入疟疾、STHs和IPIs的防治战略中。

Translated from English version into Chinese by Xin-Yu Feng, edited by Pin Yang

CDC CHINESE CENTER FOR DISEASE CONTROL AND PREVENTION
NATIONAL INSTITUTE OF PARASITIC DISEASES

Prévalence et facteurs de risque du paludisme, de la filariose et des parasitoses intestinales, isolées ou concomitantes, dans différents habitats humains au Gabon (Afrique centrale)

Noé Patrick M'bondoukwé, Eric Kendjo, Denise Patricia Mawili-Mboumba, Jeanne Vanessa Koumba Lengongo, Christelle Offouga Mbouoronde, Dieudonné Nkoghe, Fousseyni Touré, Marielle Karine Bouyou-Akotet

RÉSUMÉ

Contexte : Le paludisme, la filariose et les parasitoses intestinales sont des maladies fréquentes et souvent concomitantes dans les pays en voie de développement. Nous avons étudié la prévalence de ces infections et leurs facteurs prédictifs dans trois habitats humains différents (rural, semi-urbain et urbain) au Gabon.

Méthodes : De septembre 2013 à juin 2014, 451 individus ont été questionnés dans le cadre d'enquêtes transversales. Des échantillons de sang et de selles ont également été analysés afin de

rechercher la présence de *Plasmodium*, de filaires, de protozoaires intestinaux et d'helminthes.
Résultats : Les parasitoses intestinales (61,1 %), notamment dues à des protozoaires (56,7 %) et aux helminthes transmis par le sol (HTS, 22,2 %), prédominaient, tandis que la prévalence de *Plasmodium falciparum* (18,8 %), *Loa loa* (4,7 %) et *Mansonella perstans* (1,1 %) était plus faible. La filariose et les HTS se présentaient surtout dans les habitats ruraux mais les infections à *Plasmodium* étaient plus fréquentes dans les zones périurbaines. Les parasitoses intestinales (PI) les plus fréquentes étaient la blastocystose (48,6 %), suivie de l'ascaridiase (13,7 %), de la trichurose (11,8 %), des amibiases (9,3 %), de la giardiose (4,8 %) et de la strongyloïdose (3,7 %). Une ankylostomiase a été décelée chez un adulte en zone rurale dans la région de Dienga. Les adultes présentaient une prévalence plus élevée de *Blastocystis hominis* et de HTS, tandis que *Giardia duodenalis* était plus fréquente chez les enfants de moins de cinq ans ($P < 0,01$). Le taux de polyparasitose était de 41,5 %, avec 7,0 % de co-infections *Plasmodium*-PI et 1,8 % de *Plasmodium*-HTS. L'analyse multivariée a montré que la vie dans une zone périurbaine, le groupe d'âge de 5 à 15 ans, l'absence d'éducation ou d'éducation secondaire et la présence d'eaux de surface à proximité du domicile étaient des facteurs de risque significatifs de paludisme ($P \leq 0,01$). Pour les infestations par les HTS, les facteurs de risque identifiés étaient la consommation d'eau non traitée et la vie en zone rurale ($P \leq 0,04$). Aucun facteur prédictif significatif n'a été identifié pour les parasitoses intestinales et la co-infestation par celles-ci et le paludisme.
Conclusions : Cette étude rend compte d'une prévalence élevée des parasitoses intestinales et des protozoaires intestinaux, mais d'un faible taux de co-infestations par le paludisme et les parasitoses intestinales sur les sites de l'étude. Il serait utile d'intégrer des améliorations des conditions de vie de la population (eau saine, éducation à la santé adéquate et assainissement) dans les stratégies de lutte contre le paludisme, les HTS et les parasitoses intestinales.

Translated from English version into French by Suzanne Assenat, through

TRANSLATORS
WITHOUT BORDERS

Распространенность и факторы риска развития малярии, филяриоза и кишечных паразитов в виде отдельных инфекций или сопутствующих инфекций в различных населенных пунктах Габона, Центральная Африка

Noé Patrick M'bondoukwé, Eric Kendjo, Denise Patricia Mawili-Mboumba, Jeanne Vanessa Koumba Lengongo, Christelle Offouga Mbouoronde, Dieudonné Nkoghe, Fousseyni Touré, Marielle Karine Bouyou-Akotet

АННОТАЦИЯ

Справочная информация: малярия, филяриоз и кишечные паразитарные инфекции (КПИ) весьма распространены в развивающихся странах и часто являются сопутствующими. Распространенность и прогностические факторы развития этих инфекций изучались в трех различных видах населенных пунктов (сельских, поселковых и городских) Габона.

Методы: во время опросов, проведенных с сентября 2013 года по июнь 2014 года, было опрошено 451 человек. Кроме того, образцы крови и стула анализировали на наличие *Plasmodium*, филяриозного круглого червя, кишечных простейших и гельминтных инфекций.
Результаты: преобладали кишечные паразитарные инфекции (61,1 %), включая кишечных простейших (56,7 %) и геогельминты (ГГ) (22,2 %), тогда как *Plasmodium falciparum* (18,8 %), *Loa loa* (4,7 %) и *Mansonella perstans* (1,1 %) были менее распространены. Филярии и ГГ в основном обнаруживались в сельских поселениях, тогда как более высокий уровень распространенности плазмодиальной инфекции наблюдался в пригородных областях. Наиболее распространенным КПИ был бластоцистоз (48,6 %) с последующей частотой, в порядке убывания: аскаридиоз (13,7 %), трихуроз (11,8 %), амебиаз (9,3 %), лямблиоз (4,8 %) и стронгилоидоз (3,7 %) Анкилостома была обнаружена у одного взрослого из села Диенга. У взрослых были более распространены *Blastocystis hominis* и ГГ, тогда как *Giardia duodenalis* чаще наблюдалась среди детей в возрасте до пяти лет ($P < 0,01$). Частота случаев заражения несколькими видами паразитов составила 41,5 %, из них 7,0 % — сопутствующее инфицирование *Plasmodium* и КПИ, и 1,8 % — сопутствующее инфицирование *Plasmodium* и ГГ. Многофакторный анализ показал, что проживание в пригородной зоне, принадлежность к возрастной группе от 5 до 15 лет, отсутствие среднего образования или наличие открытого водоема неподалеку от дома являются значимыми факторами риска развития малярии ($P \leq 0,01$). Для инфекций ГГ выявленные факторы риска включали употребление неочищенной воды и проживание в сельской местности ($P \leq 0,04$). Не было выявлено значимых прогностических факторов для КПИ и сопутствующего инфицирования малярии и КПИ.
Выводы: в этом исследовании сообщается о высокой распространенности КПИ и кишечных простейших, но о низком уровне случаев сопутствующих инфекций малярии и КПИ на исследуемых участках. Улучшение условий жизни населения, например, с помощью налаженного водоснабжения, надлежащего санитарного просвещения и адекватных санитарных условий, должно быть включено в стратегии борьбы с малярией, ГГ и КПИ.

Translated from English version into Russian by Oksana Weiss, through

TRANSLATORS
WITHOUT BORDERS

Prevalencia y factores de riesgo para la malaria, filariasis y parásitos intestinales como infección única o co-infección en varios asentamientos en Gabón, África Central.

Noé Patrick M'bondoukwé, Eric Kendjo, Denise Patricia Mawili-Mboumba, Jeanne Vanessa Koumba Lengongo, Christelle Offouga Mbouoronde, Dieudonné Nkoghe, Fousseyni Touré, Marielle Karine Bouyou-Akotet

RESUMEN

Antecedentes: En los países en desarrollo, la malaria, la filariasis y las infecciones intestinales por

parásitos son comunes y a menudo se superponen. Se investigó la prevalencia e indicadores de dichas infecciones en tres asentamientos diferentes (rural, semi-urbano y urbano) en Gabón.

Métodos: En el transcurso de los estudios transversales que se llevaron a cabo entre septiembre de 2013 y junio de 2014, se entrevistaron 451 personas. Además, se analizaron muestras de sangre y materia fecal en búsqueda de infección por *Plasmodium*, gusano redondo filarial, protozoos intestinales y helmintos.

Resultados: Predominaron las infecciones intestinales por parásitos (61,1%), incluidos protozoos intestinales (56,7%) y helmintos transmitidos por el suelo (STHs) (22,2%), mientras que las infecciones por *Plasmodium falciparum* (18,8%), *Loa loa* (4,7%), y *Mansonella perstans* (1,1%) fueron menos prevalentes. La filariasis y la helmintiasis transmitida por el suelo se encontraron principalmente en los asentamientos rurales, mientras que se observó una tasa más elevada de prevalencia por infección plasmodial en las zonas periurbanas. La infección intestinal por parásitos más común fue la blastocistosis (48,6%), seguida por ascaridiasis (13,7%), tricuriasis (11,8%), amebiasis (9,3%), giardiasis (4,8%) y estrongiloidiasis (3,7%). Se detectó uncinariasis en un adulto de la zona rural de Dienga. Los adultos tuvieron una prevalencia más elevada de *Blastocystis hominis* y helmintiasis transmitidas por el suelo, mientras que la *Giardia duodenalis* se observó mucho más frecuentemente en niños menores de cinco años ($P < 0,01$). El índice de poliparasitismo fue de 41,5%, con 7,0% de infecciones intestinales por parásitos-*Plasmodium*- y 1,8% por co-infecciones *Plasmodium*-helmintiasis transmitidas por el suelo. El análisis multivariante mostró que el vivir en la zona suburbana, pertenecer al grupo etario de 5 a 15 años, sin educación o con educación secundaria, o con una masa de agua abierta cerca de la casa, fueron factores de riesgo significativos para la malaria ($P \leq 0,01$). Para las infecciones por helmintos transmitidos por el suelo, los factores de riesgo identificados fueron el beber agua no tratada y vivir en una zona rural ($P \leq 0,04$). No se identificaron indicadores de importancia para las infecciones intestinales por parásitos y la co-infección por malaria e infecciones intestinales por parásitos.

Conclusiones: El presente estudio reporta una alta prevalencia de infecciones intestinales por parásitos y protozoos intestinales, pero una tasa baja para las co-infecciones por malaria-infecciones intestinales por parásitos en los sitios de estudio. Un mejoramiento de las condiciones de vida de la población como el suministro adecuado de agua y de educación para la salud y saneamiento, deberían integrarse a las estrategias de control para la malaria, las helmintiasis transmitidas por el suelo y las infecciones intestinales por parásitos.

Translated from English version into Spanish by Maria Alejandra Aguada, through

TRANSLATORS WITHOUT BORDERS
