# Supplementary material for: Prevalence of and risk factors for malaria, filariasis, and intestinal parasites as single infections or co-infections in different settlements of Gabon, Central Africa
Source: Infect Dis Poverty. 2018 Jan 30;7:6. doi: 10.1186/s40249-017-0381-4 (PMC5789590; doi:10.1186/s40249-017-0381-4)
Supplement: Supplementary file 2 — a) Number of blood samples per site and positive results according to diagnostic methods; b) Number of stool samples per site and positive results according to diagnostic methods. (DOCX 102 kb) [file 40249_2017_381_MOESM2_ESM.docx]

Additional File 1a

Additional file 1b
